# Development and Characterization of a Genetic Mouse Model of KRAS Mutated Colorectal Cancer

**DOI:** 10.3390/ijms20225677

**Published:** 2019-11-13

**Authors:** Radhashree Maitra, Thongthai Thavornwatanayong, Madhu Kumar Venkatesh, Carol Chandy, Dov Vachss, Titto Augustine, Hillary Guzik, Wade Koba, Qiang Liu, Sanjay Goel

**Affiliations:** 1 Department of Oncology, Albert Einstein College of Medicine, Montefiore Medical Center, 1300 Morris Park Avenue, Bronx, NY 10461, USA; thongthai.thavornwatanayong@einstein.yu.edu (T.T.); Madhukumar.Venkatesh@kashivbio.com (M.K.V.); cchandy@montefiore.org (C.C.); dov.vachss@downstate.edu (D.V.); tiaugusti@montefiore.org (T.A.); 2 Department of Biology, Yeshiva University, New York, NY 10033, USA; 3 Analytical Imaging Facility, Albert Einstein College of Medicine, Bronx, NY 10461, USA; hillary.guzik@einstein.yu.edu; 4 Department of Radiology (Nuclear Medicine), Albert Einstein College of Medicine, Bronx, NY 10461, USA; wade.koba@einstein.yu.edu; 5 Department of Surgical Pathology, Montefiore Medical Center, Bronx, NY 10467, USA; qliu@montefiore.org

**Keywords:** APC, KRAS, CDX_2_, CRE, tamoxifen

## Abstract

Patients with KRAS mutated colorectal cancer (CRC) represent a cohort with unmet medical needs, with limited options of FDA-approved therapies. Representing 40–45% of all CRC patients, they are considered ineligible to receive anti-EGFR monoclonal antibodies that have added a significant therapeutic benefit for KRAS wild type CRC patients. Although several mouse models of CRC have been developed during the past decade, one genetically resembling the KRAS mutated CRC is yet to be established. In this study C57 BL/6 mice with truncated adenomatous polyposis coli (APC) floxed allele was crossed with heterozygous KRAS floxed outbred mice to generate an APC^f/f^ KRAS^+/f^ mouse colony. In another set of breeding, APC floxed mice were crossed with CDX2-Cre-ER^T2^ mice and selected for APC^f/f^ CDX2-Cre-ER^T2^ after the second round of inbreeding. The final model of the disease was generated by the cross of the two parental colonies and viable APC ^f/f^ KRAS ^+/f^ CDX2-Cre-ER^T2^ (KPC: APC) were genotyped and characterized. The model animals were tamoxifen (TAM) induced to generate tumors. Micro-positron emission tomography (PET) scan was used to detect and measure tumor volume and standard uptake value (SUV). Hematoxylin and eosin (H&E) staining was performed to establish neoplasm and immunohistochemistry (IHC) was performed to determine histological similarities with human FFPE biopsies. The MSI/microsatellite stable (MSS) status was determined. Finally, the tumors were extensively characterized at the molecular level to establish similarities with human CRC tumors. The model KPC: APC animals are conditional mutants that developed colonic tumors upon induction with tamoxifen in a dose-dependent manner. The tumors were confirmed to be malignant within four weeks of induction by H&E staining and higher radioactive [18F] fluoro-2-deoxyglucose (FDG) uptake (SUV) in micro-PET scan. Furthermore, the tumors histologically and molecularly resembled human colorectal carcinoma. Post tumor generation, the KPC: APC animals died of cachexia and rectal bleeding. Implications: This model is an excellent preclinical platform to molecularly characterize the KRAS mutated colorectal tumors and discern appropriate therapeutic strategies to improve disease management and overall survival.

## 1. Introduction

Colorectal cancer (CRC) is initiated when the colonic epithelial loses the function of the adenomatous polyposis coli (APC) pathway which is essentially a part of Wnt signaling pathway [1,2]. Loss of APC function results in nuclear accumulation of β-catenin which triggers a series of modulations to several transcription factors; an important one being TCf-4, which in turn upregulates several target genes including the cyclin D1 proto oncogene [3]. Ultimately, APC mutations affect the G1 to S transition of the cell cycle causing cell growth dysregulation and initiating the formation of intestinal polyps [4]. The APC mutant mice developed many adenomas in their small intestines and a few in the large intestine that rarely progress to invasive adenocarcinoma [1]. To overcome the drawbacks, Henoi et al. [5] developed mice (CPC; APC) carrying CDX2PNLS Cre recombinase transgene and a loxP-targeted APC allele that led to the development of primarily colorectal tumor in 17% of the mice when followed for 300 days. These CPC; APC animals resembled human colorectal lesions with biallelic APC inactivation, β-catenin dysregulation, global DNA hypomethylation, and aneuploidy. A current view is that most colorectal cancers arise from adenomatous precursors and accumulate gain-of-function mutations in proto-oncogenes and loss-of-function mutations in tumor suppressor genes resulting in the initiation of adenomatous lesions and progression to carcinoma [6,7]. The development of neoplasia is a multistep process involving the inactivation of a variety of tumor-suppressor and DNA-repair genes and simultaneous activation of certain oncogenes.

Numerous mouse models of CRC have been developed, providing insights into pathogenesis mechanisms, tools for discovery, validation of novel therapeutic targets, and a predictive platform in which to test new prevention and therapeutic strategies [1]. The orthotropic and syngeneic mice models are commonly used as a surrogate of CRC where mouse CRC cell lines harboring the KRAS mutation are used to generate a KRAS mutated CRC mimicry.

The fact that CRC patients harboring the KRAS mutation in their neoplastic tissues have detrimental effect upon administration of monoclonal antibodies like cetuximab and panitumumab, which has otherwise improved the median survival of KRAS wild type patients, has prompted the necessity to precisely understand the molecular events caused by KRAS mutation in the CRC microenvironment. To achieve this goal a genetic mouse model of KRAS mutated CRC is essential.

Moreover, based on the current knowledge, 80% of the sporadic CRC population harbor APC mutation while 45% of all CRC patients have KRAS mutation [6]. This indicates that at least 25% of CRC patients have both APC and KRAS mutations. Thus, constructing a model that carries both APC and KRAS mutations is also important for thorough molecular evaluation of the cancer. Though, it is relevant to mention that attempts to generate such models with APC inactivation and KRAS mutation has proven to be embryonically lethal thus making it impossible to have a model of KRAS mutated CRC.

In the current study we used simple strategies to develop conditional genetic mouse models where both the alleles, namely floxed APC and mutated KRAS, can be simultaneously expressed by tamoxifen, which activates the large intestine specific transcription factor CDX2 through CRE recombinase fused to mutated estrogen receptor ERT2 [8]. The more efficient and specific CRE-ERT2 can only gain access to the nuclear compartment after exposure to tamoxifen. When these mice are bred with mice containing loxP-flanked sequence, tamoxifen-inducible, Cre-mediated recombination will result in deletion of the floxed sequences in the Cre-expressing cells of the offspring. Following this strategy, we have successfully generated and molecularly characterized the model to establish resemblance to human KRAS mutated CRC. This model is thus far the most robust model to investigate the KRAS mutated CRC.

## 2. Results

### 2.1. Successful Development of Viable Mice Colony with Tamoxifen Inducible Molecular Switch

**Figure 1:**
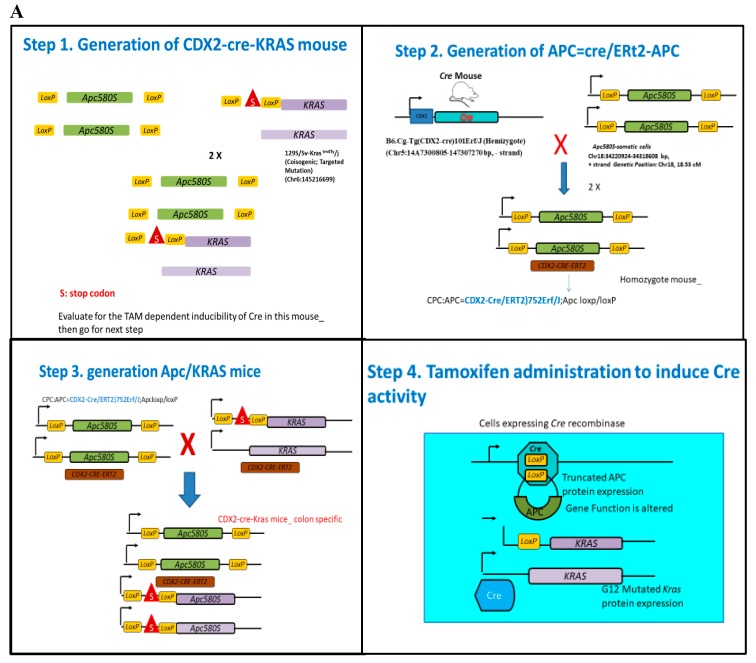

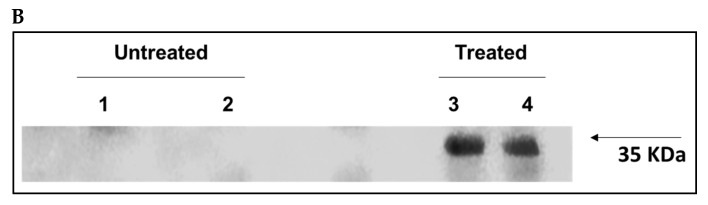
(**A**) Schematic representation of the strategy adopted for the development of the KRAS mutated CRC mice. Essentially CDX2 ERT2 Cre mice were intercrossed with mice carrying loxP-flanked adenomatous polyposis coli (APC) alleles homozygous (APC loxP/loxP, 580S) or the loxP-Stop-loxP. The final model mice with tamoxifen (TAM)-inducible KRAS G12D expression (KPC: APC) was derived by breeding a Cre^+/−^. APC ^f/f^ with KRAS ^+/-^APC ^f/f^ mice to generate APC ^f/f^ KRAS ^+/f^ CDX2-Cre-ERT2. (**B**) Western blot analysis of active KRAS pull down in untreated (1 and 2) and treated (3 and 4) KPC: APC mice (*n* = 2) shows higher KRAS activation detected in KPC: APC mice treated with tamoxifen.

Transgenic founders on a mixed background (C57BL6/J × SJL/J) were backcrossed with C57BL6/J mice in the model development. CDX2 ERT2 Cre mice were intercrossed with mice carrying loxP-flanked Apc alleles homozygous (ApcloxP/loxP, 580S) [5]. The final model harbor tamoxifen-inducible KRAS^+/−^ G12D and floxed APC (KPC APC) and was derived by breeding a Cre^+/−^ ERT2 APC ^f/f^ with KRAS^+/−^Apc^f/f^ mice (Figure 1). Genotyping for Cre ^+/−^ERT2 KRAS^+/−^ G12D mutation, and APC^f/f^ genes was done for confirmation using the primer probes (Table S4). It was observed that every animal with the desired genotype developed a tumor with tamoxifen treatment indicating that the penetrance of the transgenic mutation was 100%. The appropriate and adequate dosage of tamoxifen was determined by titration where the single dose of 0.1 mg (100 μg) of tamoxifen per 20 g of body weight (in 100 μL corn oil IP injection) was sufficient to initiate tumor generation and early mortality (Figure S1A). Tumors were detectable by positron emission tomography (PET) scan analysis on every occasion within 25–30 days of tamoxifen treatment for the experimental group while 60+ days for the positive control (Cre^+/−^ ERT2 APC^f/f^). There was no detectable tumor in the negative control (KRAS^+/−^Apc^f/f^) even after 180 days (video clips attached). The experimental group survived for an average of 165 days (*n* = 8) (Figure S1B) post tamoxifen dosage while the positive control group survived at an average of 220 days (*n* = 9). The study was terminated at 250 days. Single high dose of tamoxifen at 1 mg/20 g body weight would result in rapid initiation of tumors with survival of an average of 15 days post induction in the (KPC: APC) experimental group. At tamoxifen dosage of 100 μg per 20 g body weight the animal had an average of 24–30 days of latency before the tumor/focal lesion could be detected by PET/CT measurements. The Tamoxifen induced KPC: APC animals showed rapid disease progression during the last 25–30 days of their life (Figure S1B). Animals died with typical symptoms of rectal bleeding, significant loss of body weight, cachexia, morbidity, and particularly prominent kyphosis.

The expression of active KRAS in tamoxifen induced tumors was determined by pull down assay (*n* = 2) (Figure 1B) prior to further characterization.

### 2.2. Gross Anatomy upon Dissection

**Figure 2:**
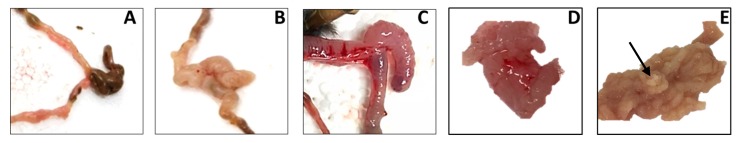
The gross anatomical appearance post tamoxifen induction in (**A**) negative control (KRAS^+/−^ and APC ^f/f^), (**B**) positive control (CDX2 CRE ERT2 and APC ^f/f^), and (**C**) model KPC: APC. (**D**) A portion of a colon of KPC: APC; (**E**) after the colon was cut longitudinally to expose the mucosa. Multiple small tumors are visible (arrow indicates one of them).

Profound inflammation of the cecum, ascending and transverse colon was observed upon tamoxifen induction in the KPC: APC experimental model (Figure 2C). Multiple small tumors were visible throughout the entire inflamed region of the colon (Figure 2D,E) when the colon was dissected longitudinally to expose the mucosal layer. Although the positive control (CDX2 CRE ERT2 and APC^f/f^) showed enlargement and inflammation of the large bowel it was to a much lesser extent than the experimental model (Figure 2B). The negative control harboring KRAS^+/−^ and APC^f/f^ with no CDX2 CRE ERT2 showed no inflammation (Figure 2A). 

### 2.3. PET-CT Scan Analysis

**Figure 3:**
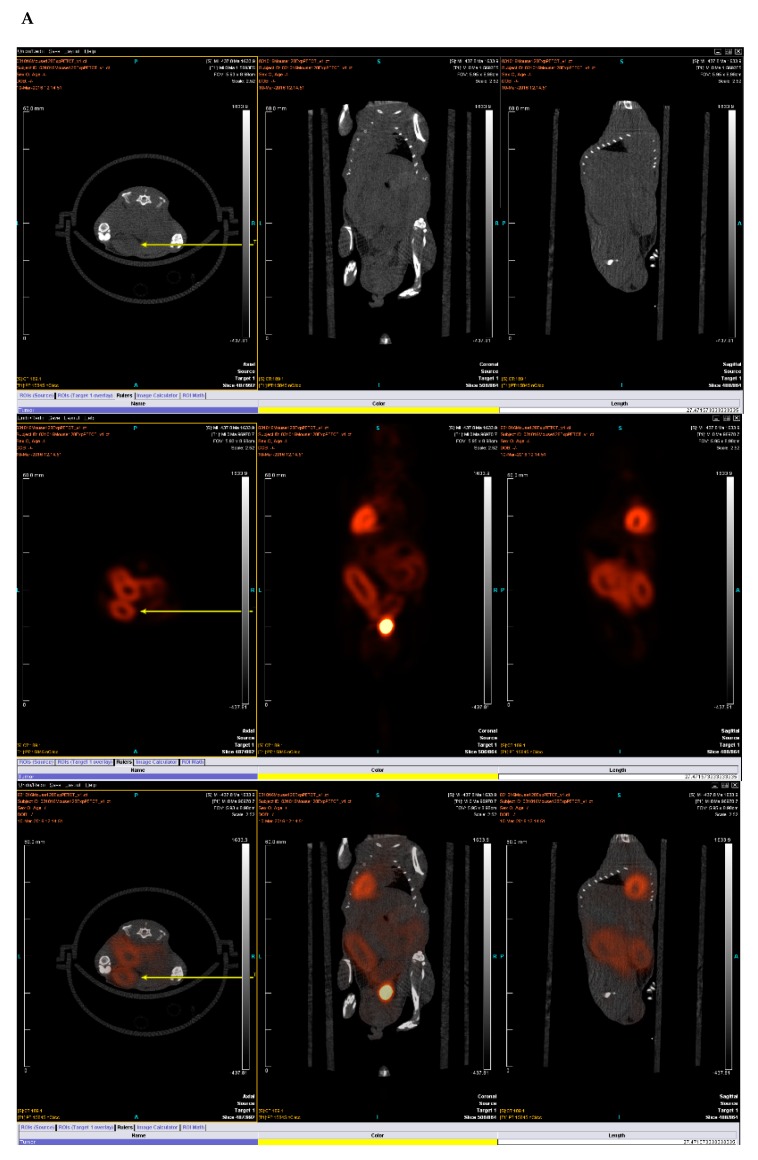

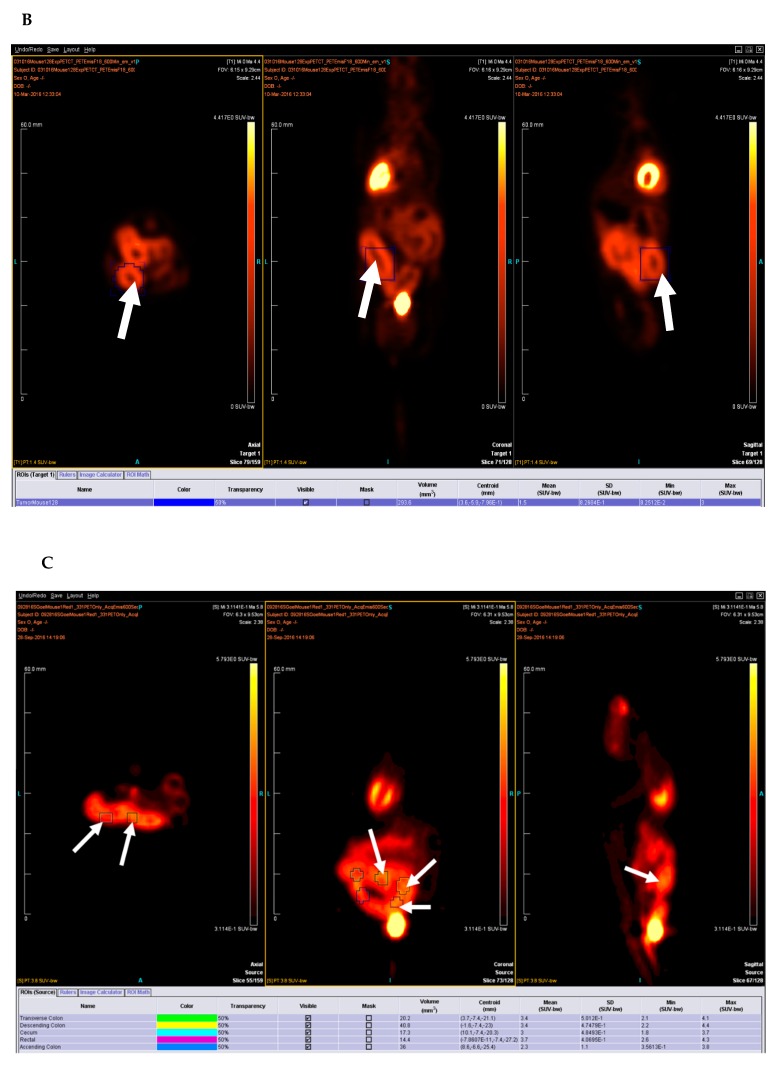
(**A**) KPC: APC animal scan analysis both by CT (top panel) and positron emission tomography (PET) scans (middle panel,) and overlay (third panel). Yellow arrows indicate the colon in transverse plane. (**B**) Depicts the standard uptake value (SUV) as recorded for a large portion of the colon of the same animal. (**C**) Shows the SUV as recorded for five different regions of the colon of a different KPC: APC animal. The white arrows indicate regions of SUV > 2.5.

Positron emission tomography and computer tomography were utilized to determine the focal tumors post 35 days of tamoxifen induction. The standard uptake value (SUV) above 2.5 was observed at several focal points (Figure 3, Figure S2 video) for KPC: APC animals confirming neoplastic lesions. Overall moderate inflammation was also observed in the positive control (Cre^+/−_^APC^f/f^) (Figure S3 video) but with no focal lesions with SUV greater than 2.5. No such inflammation was observed for the negative control (KRAS^+/−^APC^f/f^) (Figure S4 video) upon identical treatment. PET scan was repeated at *n* = 5.

### 2.4. Histopathological Analysis and Comparison to Patient Sample Biopsies

**Figure 4:**
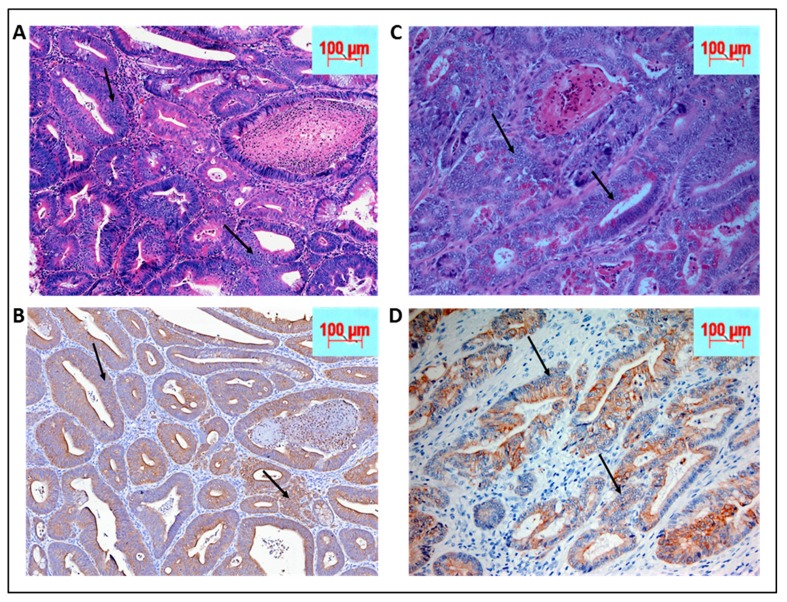
(**A**) Biopsy of KPC: APC animals post tamoxifen induction by hematoxylin and eosin (H&E) staining at 40× magnification while (**B**) shows the corresponding sections stained with pan-cytokeratin to confirm the epithelial origin of the poorly differentiated tissues. On the right panel are human biopsies obtained from Montefiore medical center repository with similar staining. (**C** and **D**) Similarities in tissue morphology of the cancer between KPC: APC animal model and human. The arrows indicate some of the many tumor regions.

Neoplasia was confirmed by our in-house pathologist (Q.L.) post hematoxylin eosin staining of the cecum and adjoining colonic tissue sectioned at 10 micrometer thickness under 20× magnification (Figure 4A,B). Since the tissue morphology was significantly lost, the mouse biopsies were immunohistochemically stained with pan-cytokeratin to confirm the epithelial origin of the poorly differentiated tumor cells. (Figure 4A,B). The mouse tumor tissue was further compared to human colorectal cancer biopsy slides obtained from the institutional repository (Figure 4C,D). The mouse colon tumor morphology was very similar to the human counterpart (Figure 4A,B compared to Figure 4C,D).

### 2.5. Determination of DNA Repair Status of the Tumor Tissue

**Figure 5:**
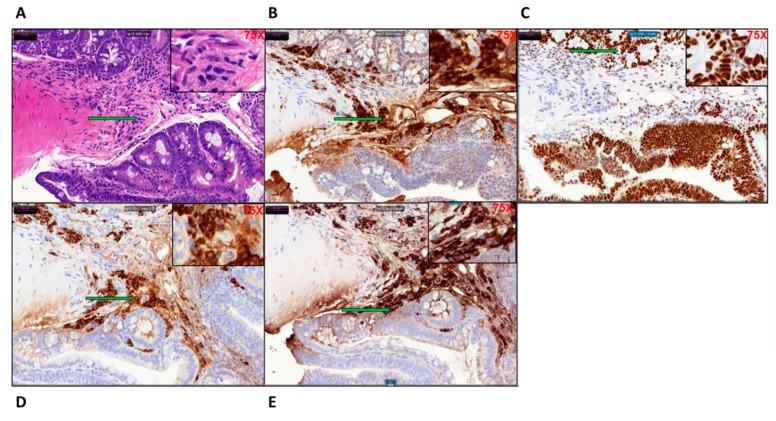
The microsatellite stable (MSS) characteristic of the KPC: APC colon cancer tissue with distinct positive staining for (**A**) H&E, (**B**) PMS2, (**C**) MSH6, (**D**) MSH2, and (**E**) MLH1; the characteristic found in 85% of human CRC at (20×) magnification (75× inset). The arrows indicate the region of the tumor tissue that has been magnified in the inset. For the control tissue staining, refer to Figure S2.

The microsatellite stability and mutational status of the DNA repair enzymes and mismatch repair genes (MMR) were determined by immune-histochemical staining of the tamoxifen induced KPC: APC colonic tissues. Like human CRC these mouse colonic tissues were microsatellite stable (Figure 5A–E) expressing H&E, PMS2, MSH6, MSH2, and MLH1. The expression of PMS2 and MSH6 was significantly higher than the other two MMR gene products MLH1 and MSH2. The staining pattern clearly indicates that the KPC: APC animals had a microsatellite stable (MSS) profile, a characteristic also found in about 85% of human CRC.

### 2.6. Molecular Characterization of the KPC: APC Tumor Tissues

**Figure 6:**
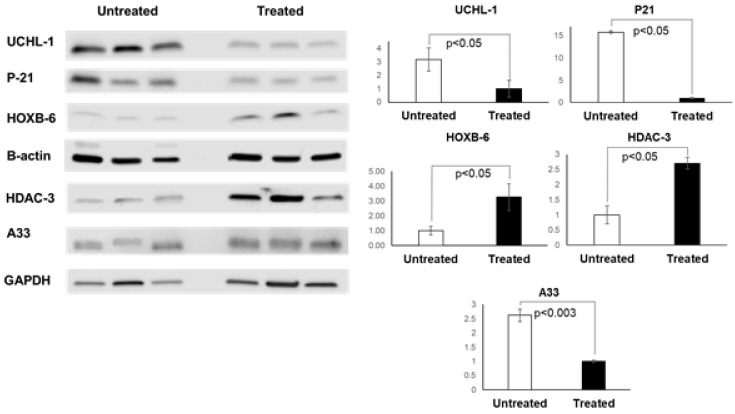
Tamoxifen treatment induced changes in expression of CRC protein markers in KPC: APC mice. (**A**) Western blot analysis of ubiquitin carboxyl-terminal hydrolase L1 (UCH-L1), P-21, HOXB-6, HDAC-3, and A33 proteins obtained from mouse tissue lysate. (**B**) Densitometry showing an average band intensity of proteins in the untreated and treated groups (*n* = 3 for each group) after normalization to the housekeeping protein (B-actin for UCHL-1, P-21, and HOXB-6, and GAPDH for HDAC-3 and A33).

Upon analysis of transcriptome sequencing data from 56 human CRC cell line (23 KRAS wild type and 33 KRAS mutant) [9] separated by KRAS mutational status showed significant upregulation (*p* = 0.0011) of home box gene HOXB6. It has been reported that altered expression of transcription factor HOXB6 can result in development of CRC; hence we determined the expression transcription factor HOXB6 in our KPC: APC animals’ pre and post tamoxifen induction. There was a significant upregulation of HOXB6 (Figure 6A,B). To further confirm that our model indeed mimics the human disease we also determined the change in expression of four more proteins namely UCH-L1, P-21, HDAC-3, and A33 which play a crucial role in the development of colon cancer.

It has been reported that P21, a cyclin-dependent kinase (CDK)-inhibitor which when down regulated in CRC is associated with poor prognosis [10]. In our model the expression of P21 is downregulated (*p* > 0.05) (Figure 6A,B) upon induction of the expression of floxed APC and KRAS by tamoxifen. Ubiquitin carboxyl-terminal hydrolase L1 (UCH-L1) is an integral part of the ubiquitin-proteasome system that plays an essential regulatory role in various cellular processes. In addition, its involvement in normal cellular functions, the alteration of proteasomal activity contributes to the pathological states of several clinical disorders, including cancer [11]. There are conflicting reports regarding upregulation [12] and downregulation [13] of UCH-L1 in colorectal cancer. Our own transcriptome analysis reveals a significant (*p* = 0.034) decrease of UCH-L1 expression in KRAS mutant cell lines as compared to wild type (approx. 60-fold). When the expression of UCH-L1 was determined by immunoblot (Western) analysis we documented a significant decrease (*p* = 0.023) in the expression of UCH-L1 upon tamoxifen induction (Figure 6A,B).

Histone deacetylase 3 (HDAC3) is prominently over-expressed in colorectal cancer both in vitro and in vivo. [14,15,16,17]. Our model animals similarly exhibit significantly higher (*p* < 0.05) expression of HDAC3 (Figure 6A,B) when induced with tamoxifen.

A33 antigen is a transmembrane glycoprotein found in both normal and cancerous colon tissues [18]. It has been reported that this antigen is heterogeneously expressed in CRC [19] and this heterogeneity might be a contributing factor in the failure of phase I/II clinical trials in therapeutic usage of monoclonal antibody against A33 during the past decade. [20] Our KPC: APC mouse tumor cells show a much lower expression of A33 antigen upon induction of cancer when compared to the expression of un-induced colonic tissues as measured by Western blot analysis (Figure 6A,B).

### 2.7. Apoptosis Pattern of the KPC: APC Tumor Tissues

**Figure 7:**
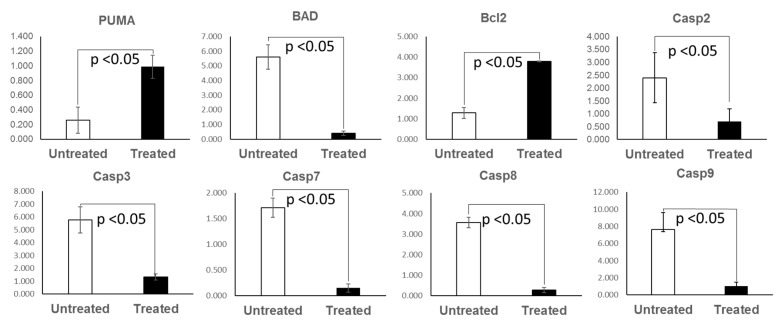
Tamoxifen treatment induced downregulation of apoptosis in KPC: APC mice. RT-qPCR analysis showing average normalized expression of apoptosis gene transcripts in the untreated and treated groups (*n* = 3 for each group). Mean numerical value of apoptosis gene transcripts expression of the negative control and tamoxifen treated groups (*n* = 3 for each group) is shown in Table S1.

**Figure 8:**
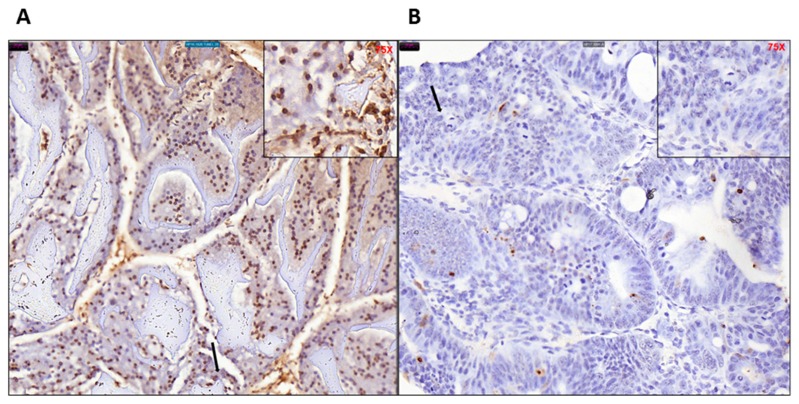
TUNEL staining of (**A**) untreated and (**B**) and treated mice colon tissues showing overall less (brown) staining in the tamoxifen-treated tissue. Difference in morphology is also evident as the untreated tissue retains normal colonic architecture while the tamoxifen treated tissue is inflamed with aggregation of tumor cells, indicated by black arrow (40× magnification, 75× inset).

Dysregulated cell proliferation, together with the obligate compensatory suppression of apoptosis is imminent to support cancer progression [21]. We performed real time QPCR analysis for a panel of pro (PUMA, BAD, caspases 2, 3, 7, 8, and 9) and anti (BCl2) apoptotic gene transcripts in tamoxifen treated and untreated colonic tissue of KPC: APC mice (*n* = 3). We observed a distinct pattern of significant (*p* > 0.05) downregulation of proapoptotic gene transcripts (total of seven specific gene transcripts) and upregulation of antiapoptotic (BCl2) gene transcript (Figure 7). The mean expression of the transcripts and the standard error of mean is shown in Table S1. To further confirm, TUNEL assay was performed with tissue samples from experimental untreated and treated groups (Figure 8A,B), and the ratio of total (blue + brown) pixel to positive (brown) pixel of the total tissue was scanned and determined (*n* = 3) to be five-fold (*p* = 0.000168), further confirming the downregulation of apoptosis upon tamoxifen induction.

## 3. Discussion

Animal models for human colon cancer can be useful for studying the mechanism of cancer development and for testing cancer prevention and treatment approaches. However, several recent reviews clearly show that the available models are flawed and the cancer that develops in these models is often significantly different from human colon cancer in terms of latency, intestinal location, or molecular signature [22]. Several animal models have been developed in the past decades namely APC*^min^* (adenomatous polyposis coli), AOM (azoxymethane), MNU (*N*-methyl-*N*-nitrosourea), MMR (mismatch repair), TGF-β, (transforming growth factor-beta) xenograft, and the non-murine zebrafish models of human colorectal cancer (CRC) disease [23]. Further, the orthotopic model has the advantage of closely mimicking human CRC including tumor microenvironment [24,25,26]. It recapitulates all of the critical components of the tumor microenvironment, as well as all of the angiogenic factors, growth factors, and cytokines, thus finely mimicking the human CRC in terms of both metastasis and microenvironments, allowing evaluation of the alterations in and modulations of the microenvironment on tumorigenesis and progression [24]. Recently one of the orthotopic models has been extended and refined into a sophisticated GEMM-derived orthotopic transplant model of KRAS-mutant colorectal cancer for high-throughput drug discovery screening and candidate drug validation [27]. This model has its own drawbacks wherein it is difficult to recapitulate the adenoma carcinoma transition to understand initiation and inception of neoplasia.

Our genetic model represents KRAS mutation and APC gene truncation, which is genetically identical to KRAS mutated CRC patients. As the onset of the cancer is tightly controlled under tamoxifen inducible CDX2 Cre, the changing etiology of the disease can be monitored in a regulated fashion which would be helpful in determining the changes at the different time points of disease progression. APC and KRAS are the most frequent mutations encountered in CRC [28] and our model can express both the mutation simultaneously along with definitive development of neoplasm.

Development of a preclinical animal model that mimics KRAS mutated colorectal cancer is urgent. Colorectal cancer patients with tumors harboring KRAS mutation are excluded from receiving the otherwise beneficial therapy of anti-EGFR monoclonal antibodies, and currently have no alternate FDA-approved treatment options available. Ongoing research requires validations of different modalities on a robust preclinical study platform that will resemble the disease phenotype and genetic profile. Heterozygous mutation of KRAS oncogene along with deletion mutation of adenomatous polyposis coli (APC) gene is embryonically lethal in animal models. In this context we have developed a viable mouse model of colorectal cancer bearing KRAS mutation that authentically serves the purpose of a preclinical model for the human CRC disease.

The final model was generated by the cross of the KRAS^+/−^ colony and CDX2Cre APC ^f/f^ colony when the desirable APC^f/f^ KRAS^+/−^ CDX2-Cre-ERT2 was developed, genotyped, and characterized. The model animals were under a tamoxifen inducible molecular switch. Micro PET scan was utilized to detect and measure tumor volume and standard uptake value (SUV). H&E staining was done to establish neoplasm and immunohistochemistry was performed to determine histological similarities with human FFPE biopsies. The MSI/MSS status was also determined. Our model tends to resemble MSS characteristics, which is prevalent in about 85% of the human CRC population. Although the human CMS3 subgroup includes patients with mutated KRAS and are characterized by a mixed MSI status [29], this group possibly represents a transition phenotype or intra-tumoral heterogeneity. Our data show strong staining for MSH6 and PMS2 but a lesser degree of staining for MLH1 and MSH2, which is probably an indication of this transition phenotype.

Due to the CDX2 tissue specific promoter characteristics, the onset of focal lesion was found to be initiated at the starting of the large bowel right at the ileocecal junction. In the early stages inflammation and focal lesion were most prominent in the cecum region and progressed to the entire colon. The rectum did not show any anomaly during the entire study. We have clearly established that the neoplasm developed in our KPC: APC model closely resembles the human CRC with distinct upregulation of HOX B6 and HDAC 3 and suppression of the expression of cyclin Kinase inhibitor P21. Similarly, the downregulation of mediators of programmed cell death that includes the initiator caspases (caspase 2, 8, 9), and executioner caspases (caspase 3 and 7) clearly support the transformed nature of the tamoxifen-induced tumors. The prominent upregulation of BCl2 and downregulation of BAD further confirms the fact. Furthermore, this model also provides the flexibility of inducing the disease at a definite time point and age fashion. Prior to induction, the animals are phenotypically normal. The CDX2-ERT2 molecular switch is very tight that we have not encountered a single animal that developed a tumor or disease phenotype without tamoxifen induction. It has not been possible to phenotypically distinguish between the positive, negative, and the experimental group without tamoxifen induction. Hence during later studies, we compared the tamoxifen treated and untreated animal within the experimental group only. The two parental strains which initially served as our positive and negative control showed limited effects with tamoxifen induction where the negative control did not develop tumors or mutant KRAS expression at the maximum dosage of tamoxifen (TAM) (1 mg/20 g body weight i.p. injection for five consecutive days) and the positive control developed a tumor with very low SUV with a prolonged period of latency (approx. 85–100 days) post tamoxifen administration at the highest dose. On the contrary our model rapidly developed aggressive KRAS mutated high SUV tumors at a very low (single injection of 100 μg) tamoxifen injection. The aggressiveness and the latency period were dependent on the concentration of tamoxifen making this model a unique tool for elucidation of the molecular mechanism of CRC. Additionally, unlike the human population, these animals have identical genetic backgrounds and can efficiently serve to analyze the complex cancer genomics under various treatment conditions similar to studies described [28] in the human population and this will further our knowledge of the disease etiology. Our model includes mutations in APC and KRAS, and therefore represents the most common genetic alterations detected in human colorectal cancer specimens. A survey of the c-bio portal dataset across seven studies encompassing 3475 samples revealed that the alterations observed included APC (61%), TP53 (54%), KRAS (35%), PIK3CA (19%), BRAF (11%), and NRAS (5%). Among these, the KRAS, NRAS, and BRAF have clinical significance.

Induction of biallelic APC loss and heterozygous KRAS mutation in our mice led to the development of cancers in the proximal colon including the cecum. A survey of the SEER dataset [30], among 180,605 patients, 31% of patients had a proximal cancer including the appendix, cecum, or ascending colon. Induction of more distal tumors including the sigmoid colon, recto sigmoid, and the rectum represent 50% of incidence in humans continues to remain a challenge. For example, in the APC: min model of biallelic APC knockout mice, the tumors observed are benign small bowel adenomas. It is fair to state, that there is a biological preference in mice to develop tumors proximally rather than distally. The early development of proximal tumors forces intervention to maximize the experimental output in the now limited life expectancy of the animal. The key may lie in gene manipulation such that the carcinogenesis is controlled at a rate that will facilitate more distal tumor development. In keeping with the above hypothesis, the introduction of a KRAS mutation combined with the controlled induction of biallelic APC loss in our model has moved the development of the tumor more distally, from the small bowel to the proximal colon, while simultaneously changing the histology from a benign adenoma to a malignant adenocarcinoma. Thus, the KPC: APC model will serve as an advanced preclinical platform for better understanding the disease mechanism at the molecular level as well as for evaluation of various therapeutics.

Our model provides a robust platform to study the intricacies of oncogenic KRAS mutation that complicates cancers like that of the colon. We have successfully developed a viable animal model of KRAS mutated colorectal cancer with tamoxifen inducible tumors harboring KRAS and APC mutations. The animal model tumors mimic the human disease type both genetically, immunohistochemically, and molecularly. This model can serve as an authentic preclinical platform for understanding the disease mechanism at the molecular level as well for evaluation of various therapeutic interventions.

## 4. Methods

### 4.1. Animal Colonies

All experimental procedures were performed after approval from the Albert Einstein College of Medicine, Animal Welfare Committee on Use and Care of Animals (IACUC, 2017206, on 13 March 2019) and according to the New York State and U.S. federal regulations. All the mice were housed in specific pathogen-free conditions within the barrier facility.

### 4.2. Animal Breeding

The final model mice with tamoxifen-inducible KRAS G12D expression (KPC APC) was derived by breeding a Cre^+/−^ APC^f/f^ with KRAS^+/−^Apc^f/f^ mice. Genotyping for Cre^+/−^, KRAS G12D mutation, and APC^f/f^ genes was done by TransnetYX using the primer probes found in Table S2. The breeding was initiated by crossing C57 BL/6 mice with truncated APC floxed allele and heterozygous KRAS floxed outbred mice to generate an APC ^f/f^ KRAS ^+/f^ mouse colony. In another set of breeding APC floxed mice were crossed with CDX2-Cre-ERT2 mice and selected for APC^f/f^ CDX2-Cre-ERT2 after a second round of inbreeding. The final model (APC ^f/f^ KRAS ^+/f^ CDX2-Cre-ERT2) was generated by crossing the two parental strains APC^f/f^ KRAS^+/f^ and APC^f/f^ CDX2-Cre-ERT2.

### 4.3. Tumor Generation

Eight to 10-week old APC^f/f^ KRAS^+/f^ CDX2-Cre-ERT2 mice were injected with 0.1 mg of tamoxifen (TAM) dissolved in corn oil (Sigma-Aldrich, St. Louis, MO, USA # T5648 and #C8267) per 20 g body weight via intraperitoneal route (i.p.) and was monitored for cancer development. Mice that reached critical condition reported by ≥10% weight loss and focal growth with standard uptake value (SUV) > 2.5 as determined by PET/CT scan, as well as the remaining mice in the experimental cohort were then sacrificed for tissue collection. Mice were euthanized using isoflurane. The cecum and the inflamed colon tissue were excised, washed in sterile double distilled water, followed by sterile 1× PBS, frozen in liquid nitrogen, and preserved at −80 °C until further analysis. The optimal dose of tamoxifen necessary to induce a tumor was determined by titrating the animals with a single dose of intraperitoneal (i.p.) tamoxifen injection at various concentrations ranging from 1 mg/20 g body weight to 1 μg/20 g body weight.

### 4.4. PET/CT Scan

#### 4.4.1. Positron Emission Tomography (PET) and Computed Tomography (CT) Methods in the Micro-PET Imaging Facility

PET/CT scans are commonly used in humans for diagnostic imaging of many conditions including cancer and inflammation [31]. Micro-PET extends this tool to small animal imaging, allowing for research applications, mostly in mice and rats [32]. [18F] fluoro-2-deoxyglucose (FDG) is a radioactive positron emitting radiotracer used in this study.

#### 4.4.2. Animal Preparation

All mice were imaged after 8–12 h of fasting, secured to the imaging palette with a breathing tube over their snout to supply 1.5% isoflurane-oxygen mixture anesthesia to continue through the imaging portion of the procedure. Each mouse was placed on a heating pad before and during scanning to maintain normal body temperature. Mice were injected with 300–400 uCi (12–15 MBq) in 0.1 mL normal saline [18F] fluoro-2-deoxyglucose (FDG) via the tail vein and imaging began 1 h after injection.

#### 4.4.3. Image Acquisition

Imaging was performed on an Inveon Multimodality scanner (Siemens Medical Solutions USA, Malvern, PA, USA), in which CT rays are generated by 80 kV peak voltage difference between cathode and tungsten target at 0.5 mA current and 200 milli second exposure time. The CT field of view was 5.5 cm by 8.5 cm with an overall resolution without magnification of 50 microns. After each acquisition, data were sorted into 3D sinograms, and images were reconstructed using a 2D-ordered subset expectation maximization algorithm. Data were corrected for deadtime counting losses, random coincidences, and the measured non-uniformity of detector response.

#### 4.4.4. Image and Data Analysis

Analysis is performed using either ASIPRO or IRW (both Siemens Medical Solutions USA, Malvern, PA, USA) dedicated software. All image studies are inspected visually in a rotating 3D projection display to examine for interpretability and image artifact. Manual regions of interest (ROI) are defined around areas of pathologic uptake and compared with co-registered CT image data. Successive scrolling through 2D slices (each 1.2 mm thick in the axial images) permits measurement of a radioactivity within defined volumes. The activity concentration within this volume divided by the activity per gram of body mass of total injected radioactivity determines the standardized uptake value (SUV).

### 4.5. Gross Anatomy Upon Dissection

The animals were euthanized by carbon dioxide asphyxiation placed on a paraffin tray in ventral position and the limbs were stretched and pinned. Incision was made vertically along the peritoneal wall to expose the abdomen. Starting at the rectum/colon, the coiled intestines was unzipped by pulling gently, using scissors and the surface of the entire intestine was photographed. Colectomy of the inflamed colon was performed and longitudinally cut open to visualize the internal morphology of the mucosa.

### 4.6. Histopathology

The dissected and opened colon was fixed in neutral buffered formalin and subjected to paraffin embedding by Swiss roll method [33]. The paraffin embedded untreated and tamoxifen treated, mouse colon sections were deparaffinized and hydrated by transferring them through the following solutions: twice in xylene for 5 min, twice in 96% ethanol, 90% ethanol, 80% ethanol, and finally double distilled water (DDW), for 3 min. Routine hematoxylin and eosin staining was performed, and histopathological evaluation of neoplastic tumor was confirmed by a pathologist (Q.L.).

Immunohistochemical staining was performed for pan-cytokeratin A1/3 (abcam Cambridge, UK # AB27988) to establish the epithelial origin of the poorly differentiated tumor tissues. Primary monoclonal antibodies against MLH1 (clone ES05, Novocastra, Leica Biosystems Newcastle Ltd., Newcastle Upon Tyne, UK, 1:30), MSH2 (clone FE11, Calbiochem, Merck KGaA, Darmstadt, Germany, 1:50), MSH6 (clone EPR3945, Epitomics Inc, Burlingame, CA, USA, 1:200), and PMS2 (clone EPR3947, Epitomics Inc, Burlingame, CA, USA, 1:200) were applied to 5-μm-thick 10% formalin-fixed, paraffin-embedded tissue sections. The sections were deparaffinized in xylene three times, 10 min each, and subsequently rehydrated through graded alcohols to distilled water. Antigen heat retrieval was performed in 1 mM EDTA (pH 9.0) for 10 min (PMS2 15 min) using a microwave oven. Next, the sections were cooled down in room temperature for 1.5 h. After rinsing in distilled water and TBS successively, sections were incubated with specific monoclonal antigen at 4 °C for 2 h. Finally, the sections were covered with streptavidin peroxidase (Dako, Santa Barbara, CA) diluted 1:100 in PBS, incubated for 30 min at 37 °C, washed three times in PBS, and stained with 3,3′-diaminobenzidine as a substrate for the peroxidase for approximately 30 min at 37 °C. Counter staining was performed using Mayer’s hematoxylin. Cells were viewed under 40× magnification in a light microscope.

### 4.7. Morphometry

Slides were scanned on the 3DHistech P250 slide scanner (SIG #1S10OD019961-01) (Budapest, Hungary). Brown stain analysis was completed on the whole piece of tissue on every slide with 3DHistech Quant Center, using the DensitoQuant module. In this module, brown stain pixels were distinguished from the rest of the tissue by color thresh-holding. The analysis of blue versus brown areas of tissue was completed in ImageJ, using the color threshold module.

### 4.8. RNA Sequence Analysis

High-throughput mRNA sequencing (RNA-seq) offers the ability to quantify genes, analyze transcriptomes, and measure transcript expression in a single assay as described elsewhere [30]. Essentially cDNA was synthesized and put to high-throughput transcriptome sequencing for the 56 CRC cell lines analyzed for this study. The 56 cells lines were separated based on KRAS status. The constitutive expression of gene of interest was computed in the two groups and the mean and standard error of mean was determined. The detailed methodology of the high throughput sequencing and the cutoff and threshold are detailed in the following reference [9].

### 4.9. Protein Isolation and Western Blot

The whole tissue lysate was collected by mincing tissue into fine pieces then grinding with a pestle and incubating for 40 min in RIPA buffer containing protease inhibitor cocktail (Sigma-Aldrich, St. Louis, MO, USA # P8340). The lysate was then centrifuged at >12,000 rpm for 1 h to remove tissue debris. For immunoblots, the protein estimation was performed by Bradford assay and 50 µg of protein lysate was loaded into each lane. Protein was resolved by SDS-PAGE electrophoresis and transferred to PVDF(polyvinylidene difluoride) membranes then probed with primary antibodies (Table S3). The blot was then incubated with HRP-conjugated secondary antibodies (Table S3) and visualized by chemiluminescence using Clarity Western ECL (enhanced luminol-based chemiluminescent) substrate (Bio-Rad, Hercules, CA, USA# 170-5061). Visualization and densitometry were done using Li-Cor Odyssey Fc imaging system. For densitometry, the band intensity was quantified using Image Studio Lite software (Li-Cor Corporate, Lincoln, NE, USA). The quantified intensity of each sample was normalized to the intensity of its respective housekeeping protein: B-actin or GAPDH.

### 4.10. Active KRAS Pull Down Assay

For active KRAS pull down assay, the tissue lysate was obtained by mincing and grinding the cecum tissue for 2 min in NP-40 lysis buffer (Cell biolabs, Inc, San Diego, CA, USA# STA-400-K) containing protease inhibitor cocktail (Sigma-Aldrich, St. Louis, MO, USA, cat. no. P8340), then centrifuged for 2 min at 14,000 rpm to removed tissue debris. Then, 4 mg of tissue lysate were used for the pull down assay according to the kit protocol (Cell biolabs, Inc, San Diego, CA, USA # STA-400-K).

### 4.11. mRNA Isolation and RT-qPCR Analyses

Total RNA was isolated and purified using Qiagen’s RNeasy Mini kit (Qiagen, Hilden, Germany#74106). RNA concentration was estimated using Nanodrop. Then, 500 ng of RNA was used to synthesize cDNA with iScript Reverse Transcription Supermix (Bio-Rad, Hercules, CA, USA# 1001708841). For qPCR, the stock of standard sample was made from a pool of cDNA collected from 17 mice. cDNA was diluted 1:25 times with sterile double distilled water and mixed with primers (Table S4) Power Up SYBR Green mix (Thermo Fisher, Waltham, MA, USA # A25777), then amplified using the cycle and condition detailed in Table S5. The amplification was performed using Bio-Rad CFX96 Real Time System (Hercules, CA, USA). The primers used in the experiment were obtained from Millipore Sigma (Burlington, MA, USA). The GAPDH primer (Table S4) was amplified and used as a housekeeping gene for normalization.

### 4.12. TUNEL Assay

Mouse colon sections were stained in duplicate using ApopTag Plus Peroxidase in Situ Apoptosis Detection Kit (Millipore, Burlington, MA, USA# S7101). The paraffin embedded mouse colon sections were deparaffinized and hydrated by transferring them through xylene to ethanol washings. Samples were pretreated with proteinase K (20 μg/mL), quenched with hydrogen peroxide, and incubated with equilibration buffer. After washing, samples were incubated with TdT enzyme at 37 °C for 1 h, then incubated with anti-digoxigenin conjugate for 30 min at room temperature and washed 4 times with PBS for 2 min each afterward. After staining, samples were developed with peroxidase substrate, washed, counterstained, then mounted onto glass slides.

### 4.13. Statistical Analysis

Mean of data collected from 3 mice was generated for the tamoxifen treated group and untreated group in Western blot densitometry and RT-qPCR analysis. Two tailed *t*-test was used to determine statistical significance (*p* < 0.05). Statistics were calculated using Microsoft Office Excel.

## Supplementary Materials

Supplementary materials can be found at https://www.mdpi.com/1422-0067/20/22/5677/s1.
