# Supplementary material for: Development and Characterization of a Genetic Mouse Model of KRAS Mutated Colorectal Cancer
Source: Int J Mol Sci. 2019 Nov 13;20(22):5677. doi: 10.3390/ijms20225677 (PMC6888417; doi:10.3390/ijms20225677)
Supplement: Supplementary file 1 [file ijms-20-05677-s001.zip › ijms-625511-supplementary - xml - 1/ijms-625511-supplementary.pdf]

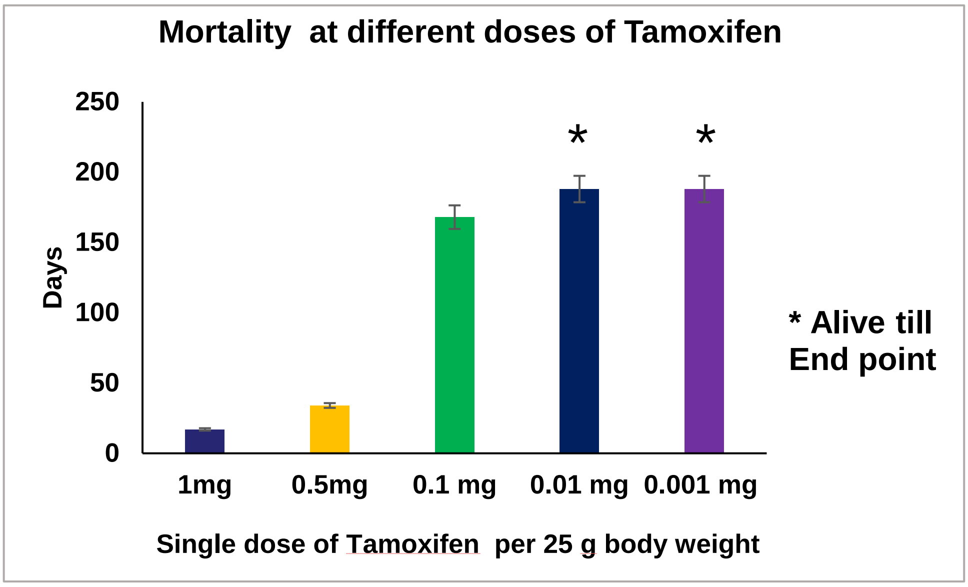

A

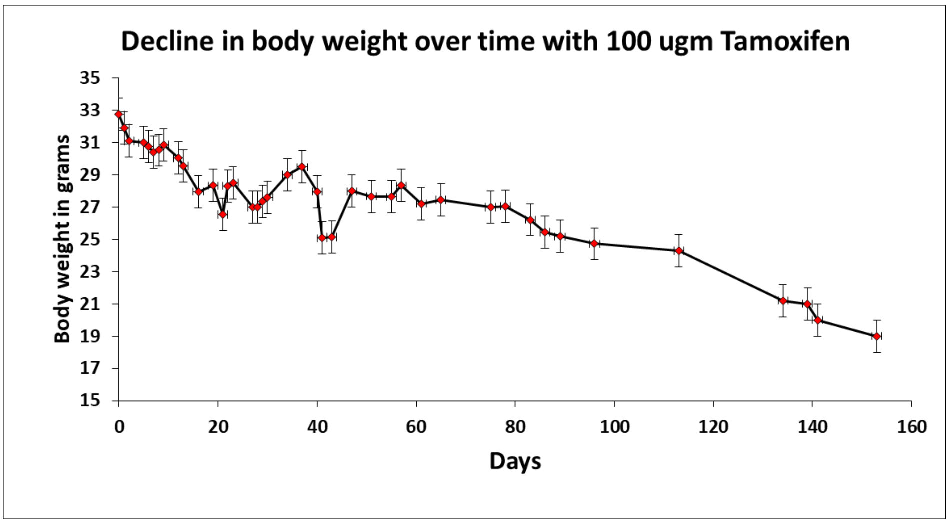

B

Figure S1. (**A**) Titration of KPC: APC animals at different dose of Tamoxifen. * indicates that the animals were alive till the end point of the study. (**B**) Is the decline of body weight of KPC: APC mice treated with a single dose of 100 microgram of tamoxifen. The results represent a mean ($n = 3$) animals.

A B C

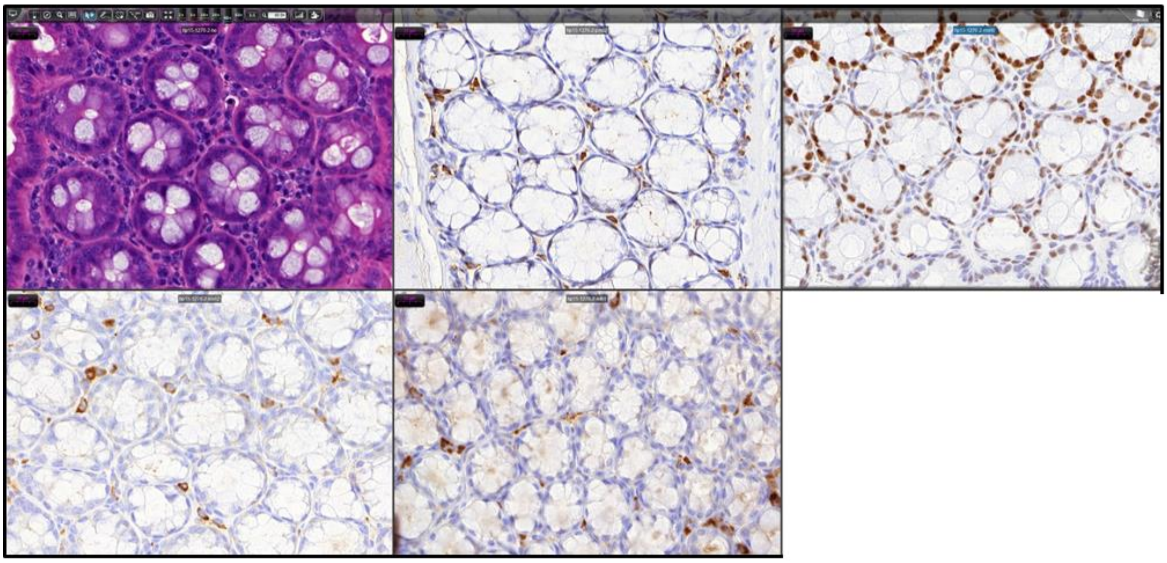

D E

Figure S2: Control mouse tissue staining for (**A**) H & E, (**B**) PMS2, (**C**) MSH6, (**D**) MSH2 and (**E**) MLH1. No prominent nuclear staining has been observed. The tissue architecture is also intact (40×).

Figure S3. PET scan of CDX2 CRE ERT2 and APC f/f (video) showing much lesser degree of overall inflammation and tumor growth but with low SUV (<2.5).

Figure S4. KRAS+/- and APC f/f (video) post TAM induction showing normal colonic architecture.

Table S1. Mean numerical value of apoptosis gene transcripts expression of the negative control and tamoxifen treated groups (*n* = 3 for each group).

		PUMA	BAD	Bcl2	Casp2	Casp3	Casp7	Casp8	Casp9
Untreated	Mean	0.259	5.600	1.282	2.397	5.764	1.714	3.560	7.624
	SEM	0.179	0.842	0.261	1.376	1.006	0.183	3.051	1.983
Treated	Mean	0.987	0.409	3.794	0.704	1.334	0.152	0.273	1.014
	SEM	0.157	0.137	0.024	0.679	0.239	0.079	2.272	0.466

Table S2. List of Primer probes used in genotyping service by Transnetxy.

Probe	Sequence (5′-3′)
KRAS G12D-F	GGCCTGCTGAAAATGACTGAGTATA
KRAS G12D-R	CTGTATCGTCAAGGCGCTCTT
Cre-F	TTAATCCATATTGGCAGAACGAAAACG
Cre-R	CAGGCTAAGTGCCTTCTCTACA
Apc Fl/Fl-F	CTGTATCATGGAAAGATAGGTGGTCATT
Apc Fl/Fl-R	GTGGTCAAGCTCGATAACTTCGTAT

Table S3. List of antibodies used for western blot analysis.

Antibodies	Company	Catalogue no.	Clone	Dilution
UCH-L1 (C-4)	Santa Cruz	sc-271639	C-4	1:200
P-21	Santa Cruz	sc-6246	F-5	1:200
HOX6B	Santa Cruz	sc-166950	B-12	1:100
HDAC-3	Santa Cruz	sc-11417	H-99	1:200
A33	Santa Cruz	sc-398702	E-8	1:200
B-actin	Millipore Sigma	A3853	AC-40	1:10000
GAPDH	Cell Signaling	5174P	D16H11	1:5000
Anti-mouse IgG-HRP linked	Cell Signaling	7076S	-	1:2000
Anti-rabbit IgG-HRP linked	Cell Signaling	7074S	-	1:2000
Mouse IgGk BP-HRP	Santa Cruz	sc-516102	-	1:5000

Table S4. List of primers used for RT-qPCR analysis.

Oligo Name	Tm (°C)	Sequence (5′-3′)
GAPDH-F	66.3	TCACCACCATGGAGAAGGC
GAPDH-R	66.3	GCTAAGCAGTTGGTGGTGCA
PUMA-F	65.6	CGGCCCAGCCTGTAAGATAC
PUMA-R	61.3	TTACAGGTAGTGCCAGATGCT
Apaf1-F	59.7	AAAGGAATGCAAAGGTTCTC
Apaf1-R	58	GATGTCTCTCTGAGCATTTC
BAD-F	58.7	CCTTCAAGGGACTTCCTC
BAD-R	57.1	TGTGGAGCAGAAGATCAC
Bax-F	60.2	CCTTTTTGCTACAGGGTTTC
Bax-R	56.5	ATATTGCTGTCCAGTTCATC
Bcl-F	53.6	ATGACTGAGTACCTGAACC
Bcl-R	57.1	ATATAGTTCCACAAAGGCATC
Casp2-F	58.4	ACAGGAAATGCAAGAGAAAC
Casp- R	57	TTTTGGCTTGTTCTGTAGAC
Casp3-F	57.3	CATAAGAGCACTGGAATGTC
Casp3-R	59.5	GCTCCTTTTGCTATGATCTTC
Casp7-F	59.2	CAAAACCCTGTTAGAGAAACC
Casp7-R	57.5	CCATGAGTAATAACCTGGAAC
Casp8-F	54.9	CAAGAGAACAAGACAGTGAG
Casp8-R	55	ACTCAGAGCCTCTTTATCAC
Casp9-F	60.4	TGATCGAGGATATTCAGCAG
Casp9-R	58.2	CCTCTAAGCAGGAGATGAAG

Table S5. Conditions and cycles used for RT-qPCR analysis.

Step	Temperature	Duration	Cycles
UDG activation	50 °C	2 minutes	Hold
Dual-Lock DNA polymerase	95 °C	2 minutes	Hold
Denature	95 °C	15 seconds	40
Anneal/extend	60 °C	1 minutes	
